# An implementation framework to improve the transparency and reproducibility of computational models of infectious diseases

**DOI:** 10.1371/journal.pcbi.1010856

**Published:** 2023-03-16

**Authors:** Darya Pokutnaya, Bruce Childers, Alice E. Arcury-Quandt, Harry Hochheiser, Willem G. Van Panhuis

**Affiliations:** 1 University of Pittsburgh, Department of Epidemiology, Pittsburgh, Pennsylvania, United States of America; 2 University of Pittsburgh, Department of Computer Science, Pittsburgh, Pennsylvania, United States of America; 3 University of Pittsburgh, Department of Biomedical Informatics and Intelligent Systems Program, Pittsburgh, Pennsylvania, United States of America; The University of Melbourne, AUSTRALIA

## Abstract

Computational models of infectious diseases have become valuable tools for research and the public health response against epidemic threats. The reproducibility of computational models has been limited, undermining the scientific process and possibly trust in modeling results and related response strategies, such as vaccination. We translated published reproducibility guidelines from a wide range of scientific disciplines into an implementation framework for improving reproducibility of infectious disease computational models. The framework comprises 22 elements that should be described, grouped into 6 categories: computational environment, analytical software, model description, model implementation, data, and experimental protocol. The framework can be used by scientific communities to develop actionable tools for sharing computational models in a reproducible way.

## Introduction

Computational models have become valuable tools for the global response against infectious disease outbreaks and pandemics, including the Coronavirus Disease 2019 (COVID-19) pandemic [1]. Computational models of infectious diseases are representations of biological phenomena in computer code, used to elucidate mechanistic processes of infectious diseases such as transmission and pathogenicity, to study the effect of countermeasures and to forecast epidemic trajectories [2–4]. As in all scientific research, the validity of a computational model depends on the ability of the community to review and reproduce the modeling “experiment” (i.e., to obtain scientific results consistent with a prior study using the same experimental methods) [5,6].

Reproducibility can be especially limited for computational studies such as computational modeling and artificial intelligence, due to their methodological complexity and heterogeneity of, or restricted access to, data sources [7,8]. The rapid pace of modeling research during the COVID-19 pandemic has raised concerns about the transparency and reproducibility of modeling results [9]. Lack of reproducibility can have serious consequences, as illustrated by retractions of early COVID-19 research from prominent scientific journals and can potentially reduce societal trust in science and, consequently, in the public health response against the pandemic [10–12].

Scientific and government agencies, including the US Government Accountability Office and the US National Academies of Sciences, Engineering, and Medicine (NASEM), have published recommendations to enhance the reproducibility of computational research [6,13]. Most recommendations list general principles or suggest types of information that should be reported by scientific publications of computational modeling experiments, such as the checklist recommended by the EPIFORGE 2020 guidelines [5,14]. The EPIFORGE checklist does not include specific items (e.g., names, versions, and dependencies) related to the computational environment or analytical software; instead, there is a general “fully document the methods” item in the Methods section. Most guidelines recommend that researchers describe the data sources and modeling methods used and that they share the source code [5,13]. Yet, even with available input data and source code, published results of modeling studies can be difficult or impossible to reproduce [6]. It is notoriously difficult to rerun a published computational model, even when source code is available, because other essential details such as the information about versions and dependencies of the software, or about the required operating system and compute environments, are often missing [15]. The contributions of our work to the current recommendations and guidelines are the improved comprehensiveness of the conceptual framework, the added specificity of the framework elements, and the iterative testing process with published infectious disease computational modeling studies.

Various initiatives have emerged in the scientific community to improve scientific inference based on computational models and to improve public health decision-making during outbreaks. Several initiatives have developed methods for comparing results across multiple models. For example, multi-model comparison studies have been conducted for rotavirus and dengue to better understand the effects of vaccination [16,17]. Recently, multi-model comparison studies for influenza and COVID-19 forecasting have evolved into coordinated projects, such as FluSight and the COVID-19 Forecast Hub, with their own infrastructure for data and model sharing to support decision-making by national and global health agencies (S1 Table). New methodology has also been developed to combine results from multiple models into ensemble results to improve epidemic forecasting, e.g., for influenza, Ebola, and COVID-19 [18–20]. In addition, formal decision-analytic methods have been developed to better characterize uncertainty in multi-model comparison projects [21].

The success and impact of model comparison and combination projects depend on methods and technologies that enable researchers to share their modeling experiments in a transparent and reproducible way that characterizes model similarities, discrepancies, and uncertainties. Thus far, the various model combination projects and modeling hubs have been dependent on ad hoc methods to describe models and share results. No methodological framework currently exists that can directly be translated into tools for sharing computational models of infectious diseases in a transparent and reproducible manner.

## Results

**Fig 1:**
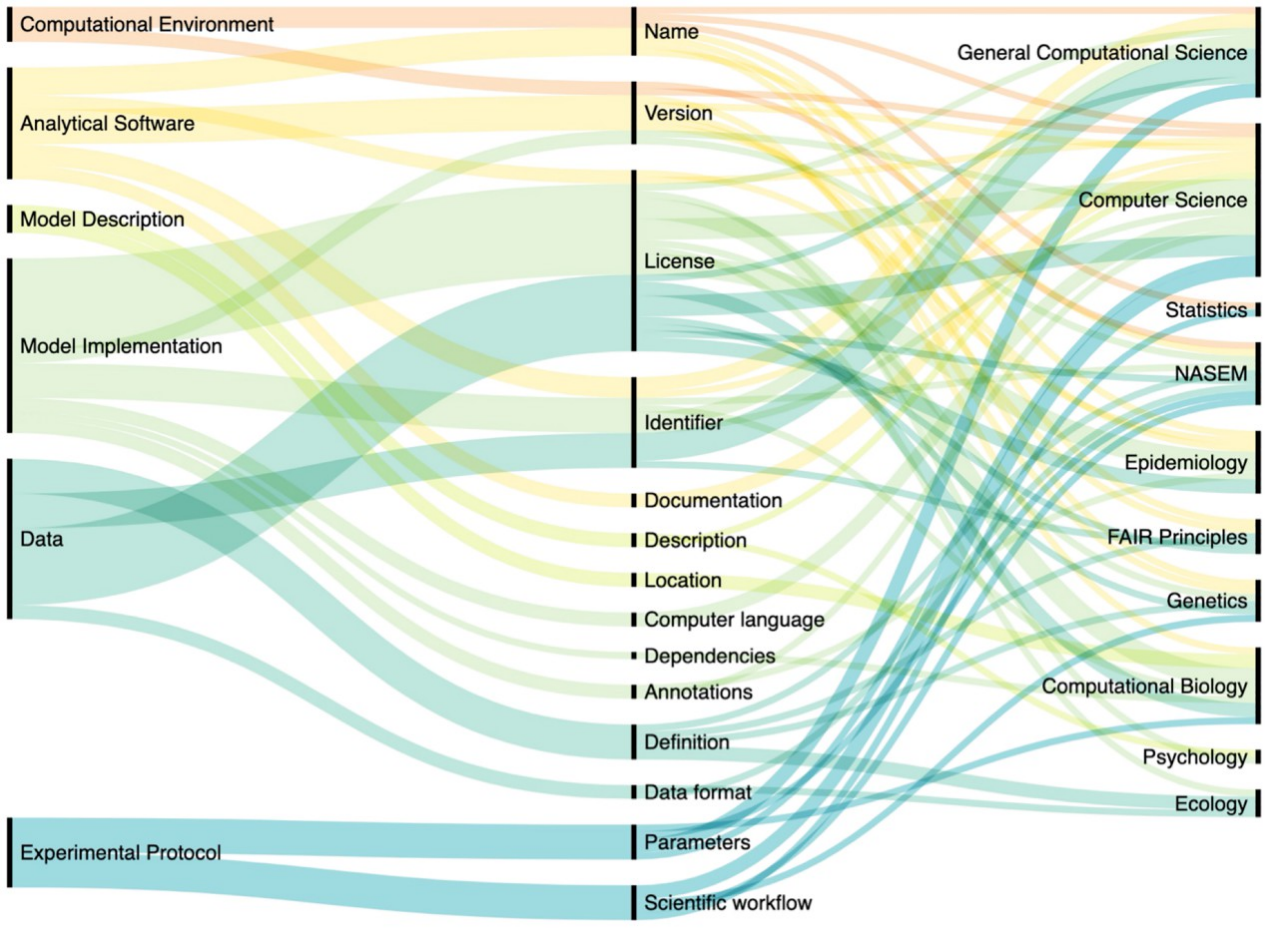
Flow diagram depicting the 6 implementation framework reproducibility categories (left), their associated elements (center), and the scientific disciples from which references were identified (right). Width of the lines are proportional to the number of references gathered from each scientific disciple to justify the inclusion of each category and element. Question 5.1, “Does the model in the publication use input data?” is omitted from the figure.

We developed an implementation framework for representing computational models of infectious diseases in a reproducible format, grounded in previous research on reproducibility from a broad range of scientific disciplines (Fig 1). The framework can be used by researchers and scientific organizations to develop tools and resources, such as checklists and metadata schemas to share transparent and reproducible computational models.

**Fig 2:**
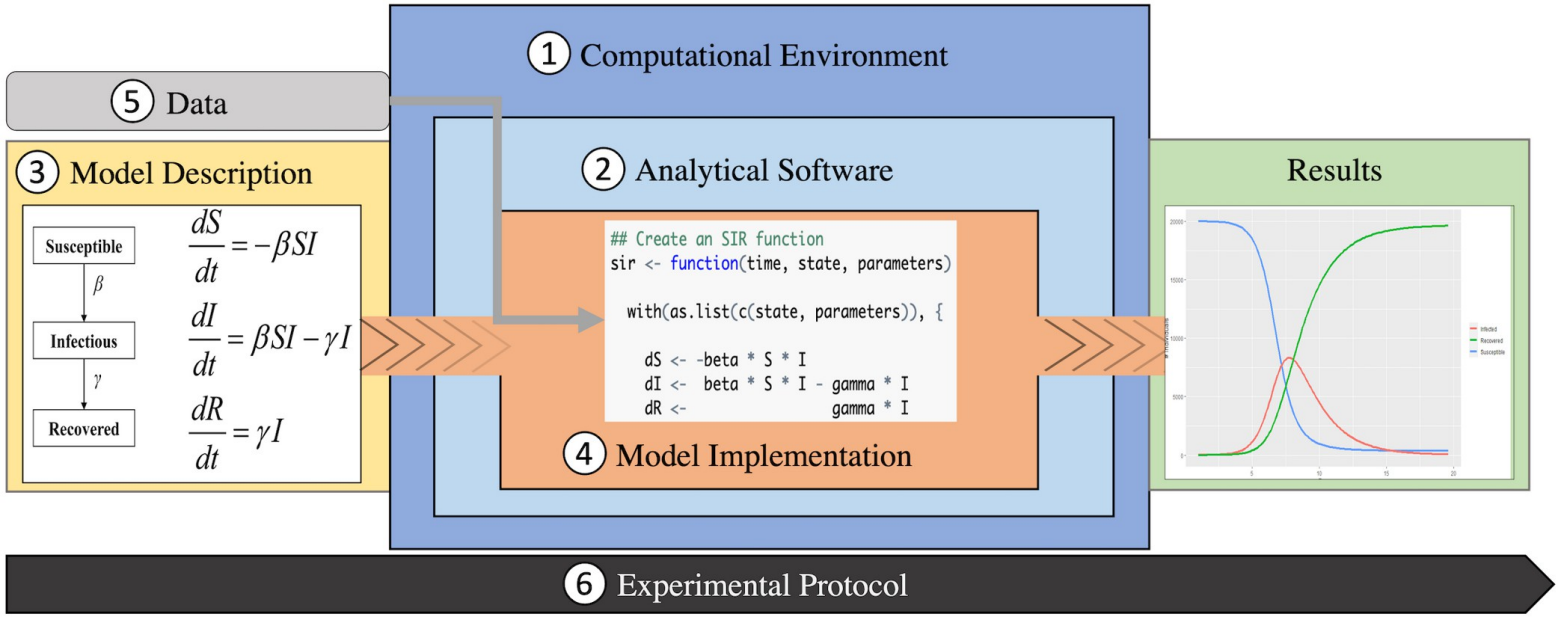
Reproducibility framework. The categories of the reproducibility framework are depicted for the example of a susceptible-infectious-recovered (SIR) model. The SIR model is described using equations in a model description (3) and implemented using a model implementation in the R language (“code”) (4). The code runs in an analytical software, in this case R (2), which runs in some computational environment with an operating system (1). The model implementation can import data (5) and operate on it. The model implementation produces results in the form of new data or visualizations that leave the analytical software (e.g., as PDF) and/or the computational environment (e.g., as printout). The experimental protocol describes the entire workflow and how the categories interact (6).

We identified 22 elements in 6 categories that together provide a complete representation of the reproducibility for a computational model (Table 1), based on a review of existing guidance on reproducibility and an iterative testing process by our team. The testing process identified discrepancies between checklist responses from different team members after reviewing the same set of infectious disease modeling papers and helped improve the robustness and consistency of the framework. The 6 categories are as follows: (1) computational environment; (2) analytical software; (3) model description; (4) model implementation; (5) data; and (6) experimental protocol. We represented the 6 categories in a framework that aligns with a commonly used workflow for computational experiments (Fig 2).

**Table 1:** Implementation framework categories, elements, and relevant examples.

Category and Element	Abbreviated Definition*	Examples
**1) Computational Environment**		
1.1) Name	Name of operating system	“Microsoft Windows,” “macOS,” “Linux”
1.2) Version	Version of operating system	“Windows 8.1 (Blue),” “macOS 10.12 (Sierra)”
**2) Analytical Software**		
2.1) Name	Name of software program	“SAS,” “R,” “original software name”
2.2) License	Restriction on use	Open source: “R”; propriety: “STATA,” “SAS”
2.3) Version	Version of software	“SAS 9.4,” “R version 3.3.3”
2.4) Identifier	Unique online identifier	“DOI,” “URL”
2.5) Documentation	Availability of documentation to use and install software	URL to installation guide
**3) Model Description**		
3.1) Description	Complete, structured description of the model	Equations, diagrams, charts, tables vs. unstructured text
3.2) Location	Specified in the publication/supplement	Model described in the methods/supplement vs. referenced in preceding publication
**4) Model Implementation (“Code”)**		
4.1) License	Restriction on use	Publicly stored on GitHub
4.2) Version	Version of code	Version, modification date
4.3) Identifier	Unique online identifier	“DOI,” “URL”
4.4) Computer language	Name of code language	“SAS,” “R,” “STATA,” “Python,” “C++”
4.5) Dependencies	Additional essential files	Packages, classes, supplementary files
4.6) Annotations	Sufficient, user-interpretable comments	Code suitably annotated for understanding
**5) Data**		
5.1) Data calibration	Indicator for whether the model was calibrated to existing vs. simulated data	“Yes” (model calibrated to input data); “no” (model does not require input data)
5.2) Definition	Description of data content and source	Content: column/field descriptions; source: “CDC,” “Project Tycho”
5.3) Identifier	Identifier from where the data are retrieved	“DOI,” “URL”
5.4) License	Restriction on use	Publicly stored on GitHub
5.5) Data format	Formatted data for the model implementation	“CSV,” “JSON,” “XML”
**6) Experimental Protocol**		
6.1) Parameters	Description of model parameters	Table or list of parameter values
6.2) Scientific workflow	Process of how categories 1–5 create the results	Explanation in GitHub README.md

*Complete definitions provided in S1 Text.

The computational environment comprises the combined software and hardware used to conduct the experiment. The analytical software includes the software, e.g., R or Python, used for the computational analysis. The computational model is usually described as a combination of narrative text, diagrams, and mathematical equations (model description). The model is then represented in its computational format as a set of commands, functions, and operations encoded in the model source code (model implementation). The data are ingested by the model implementation and operated on to compute the model results. The entire computational experiment is documented in the experimental protocol. A complete description of all the elements will represent a reproducible computational model.

## Discussion

The implementation framework provides a foundation that can be further developed by scientific communities into tools for sharing computational models of infectious diseases in a reproducible way, based on community-specific preferences. For example, the framework can be formalized into a structured metadata schema with prescribed structured vocabularies and ontologies that could render a machine-actionable metadata object compliant with the Findable, Accessible, Interoperable, and Reusable (FAIR) guiding principles [22–24]. Machine-actionable metadata could enable automated workflows for model comparison and combination efforts and accelerate the use of models for time-sensitive decision-making, e.g., in the context of a distributed ecosystem of FAIR data and services, as recently described by Bourne and colleagues [25]. Beyond just sharing computational models, machine-actionable metadata could become components of dynamic data management and sharing plans (DMSPs) for computational studies, which could streamline data and model sharing and scientific discoveries [26].

The implementation framework can also be developed into a checklist to represent the degree to which a computational modeling experiment is reproducible, as we did as part of the iterative testing process during the development of the framework (S2 Table). Similar checklists are used to assess the degree of participation of open science at research institutions or the degree of compliance with the FAIR guiding principles [27,28].

Another highly promising method for sharing computational models in a transparent and reproducible manner is through containerization in executable workflow objects, such as Galaxy [29], Open Curation for Computer Architecture Modeling (OCCAM) [30], and Pegasus [31]. Containerized experiments are especially useful when connected to scholarly publications to ease access, simplify reproduction, and build on prior experiments for new insights. For example, in a pilot project, OCCAM was connected to the Association for Computing Machinery’s (ACM) Digital Library to demonstrate how containerized workflows can be included and distributed in an executable form with a scholarly article [32]. The implementation framework described in this article specifies what information should be represented in the metadata for containerized workflow objects, so that researchers (and machines) can understand what a certain containerized object represents.

The lack of reproducibility for computational models of infectious diseases can undermine the scientific credibility of modeling results among researchers, policymakers, and even among the public, where skepticism regarding scientific research is already on the rise [33]. It is essential to represent computational models in a transparent and reproducible way so that models can be shared, compared, and combined. We envision that researchers, journals, funders, and scientific organizations can use our framework to develop actionable tools to improve sharing of computational models in a reproducible manner that also accelerates the model-to-decision timeline in response to emerging infectious disease threats.

## Methods

To identify the 6 categories and 22 elements of the implementation framework, we reviewed guidelines published by the NASEM, the FAIR guiding principles, and peer-reviewed literature in a variety of scientific domains including general computational science, computer science, statistics, epidemiology, genetics, computational biology, psychology, and ecology (Fig 1).

We identified peer-reviewed literature by querying PubMed articles published between January 1, 2000 and January 1, 2020, using keywords “reproducible,” “reproducibility,” “computational,” “research,” “data,” and “code.” We limited our search to studies published in English and excluded papers about animal-models or clinical research. We extracted quotes that referenced information that researchers believed was relevant to represent reproducible and transparent computational modeling studies.

We grouped the 22 elements into 6 categories and mapped the relationships between each of the categories to the conceptual model of an infectious disease modeling workflow (Fig 2). The workflow identifies how the 6 categories are used together to generate the model results. The final implementation framework, along with abbreviated definitions, and relevant infectious disease computational modeling study examples are presented in Table 1.

To validate the implementation framework categories and elements, we structured the framework into a checklist (S2 Table). The checklist was trialed 3 times using 10, 20, and 48 infectious disease modeling studies with varying complexities. DP completed the checklist during the first 2 trials. During the third trial, DP, BC, AAQ, and WVP completed the checklist independently. The results of all 3 trials were reviewed by the entire team. Based on discrepancies between answers, we modified the checklist to improve clarity and then moved to the next trial. Iterating through the checklists versions of the implementation framework has improved the real-world application potential of the framework, versus a more theoretical approach that may not be readily implemented by communities.

For the first trial, 10 publications were randomly selected without replacement from all publications authored by Models of Infectious Disease Agent Study (MIDAS) members before September 20, 2019 (*n* = 3,664). Based on title and abstract review, if the paper was not related to infectious disease modeling, we randomly selected another paper until we identified 10 infectious disease modeling studies. For the second trial, we randomly selected 20 COVID-19 modeling papers without replacement from a list of 229 papers authored by MIDAS members between January 1, 2020 and March 31, 2020.

For the third trial, we identified 48 publications (S3 Table). We queried PubMed, medRxiv, bioRxiv, and arXiv for COVID-19 modeling publications published between January 1, 2020 and March 31, 2020, using keywords “coronavirus,” “COVID-19,” SARS-Cov-2,” “estimate*,” “model*,” and “reproduc*.” The initial query identified 793 records with 10 duplicates. The titles and abstracts of the 783 de-duplicated publications were reviewed with inclusion and exclusion criteria. We excluded 224 records based on exclusion criteria: observational, genomic, immunological, and molecular studies, commentaries, reviews, retraction, letter to editor, response articles, not related to COVID-19, clinical trials, and app development. From the remaining 559 papers, we randomly selected 50 publications without replacement for full-text review with inclusion and exclusion criteria. A letter to the editor and review paper were excluded. Forty-eight publications were included in the final review process. During the final trial, we randomly assigned team members to each of the 48 publications; each publication was reviewed twice. After the final review, discrepancies between each response were discussed among the authors, and final edits to the implementation framework and checklist were made.

## Supporting information

S1 Text Supplementary information. Complete definitions of the implementation framework categories.
(DOCX)

S1 Table Examples of multi-model comparison initiatives for influenza and COVID-19 forecasting. (DOCX)

S2 Table Reproducibility framework formatted as a checklist with examples. The checklist consists of questions related to the 6 categories: (1) computational environment; (2) analytical software; (3) model description; (4) model implementation; (5) data; and (6) experimental protocol. The center column provides examples for each category and element.
(DOCX)

S3 Table DOI and publication title of the 48 papers assessed using the infectious disease modeling reproducibility checklist during the third trial. (DOCX)
