# Supplementary material for: An implementation framework to improve the transparency and reproducibility of computational models of infectious diseases
Source: PLoS Comput Biol. 2023 Mar 16;19(3):e1010856. doi: 10.1371/journal.pcbi.1010856 (PMC10019712; doi:10.1371/journal.pcbi.1010856)
Supplement: S1 Table — (DOCX) [file pcbi.1010856.s002.docx]

| Collaborative project | Description | URL |
| --- | --- | --- |
| FluSight | Community-based influenza forecasting effort managed by the CDC that combines influenza forecasting models. | https://www.cdc.gov/flu/weekly/flusight/index.html |
| COVID-19 Forecast Hub | Community-based effort for COVID-19 forecasting with a central repository for short-term (1-4 weeks) forecasting data from over 50 research groups. | https://covid19forecasthub.org |
| Multi-Model Outbreak Decision Support (MMODS) | Community-based decision-analytic approach established by the MIDAS community to combine and contrast results from multiple COVID-19 models. | https://midasnetwork.us/mmods/ |
| COVID-19 Scenario Modeling Hub | Modeling hub established to compare and combine models with the objective to project the course of the pandemic in the US several months ahead. | https://covid19scenariomodelinghub.org |
| Flu Scenario Modeling Hub | Modeling hub established to compare and combine models with the objective to project the course of the seasonal flu pandemic in the US several months ahead. | https://fluscenariomodelinghub.org |
| COVID-19 Multi-model Comparison Collaboration (CMCC) | Global effort between modelers and policy makers to compare and interpret COVID-19 models with a focus on models used in developing countries. | https://www.strath.ac.uk/research/subjects/managementscience/healthsystems/thecovid-19multi-modelcomparisoncollaborationcmcc/ |
| European Covid-19 Scenario Hub | European effort supporting the development and combination of short and medium-term COVID-10 models | https://covid19scenariohub.eu/ |
| US Center for Forecasting and Outbreak Analytics (CFA) | Center established by the CDC to help improve outbreak prediction and response in the US. | https://www.cdc.gov/forecast-outbreak-analytics/index.html |
| WHO Modeling Hub in Berlin | Center established by the WHO to help improve outbreak prediction and response globally. | https://pandemichub.who.int/ |
