# Supplementary material for: An implementation framework to improve the transparency and reproducibility of computational models of infectious diseases
Source: PLoS Comput Biol. 2023 Mar 16;19(3):e1010856. doi: 10.1371/journal.pcbi.1010856 (PMC10019712; doi:10.1371/journal.pcbi.1010856)
Supplement: S3 Table — (DOCX) [file pcbi.1010856.s004.docx]

|  | DOI | Publication title |
| --- | --- | --- |
| 1 | http://arxiv.org/abs/2002.06563v1 | Epidemic analysis of COVID-19 in China by dynamical modeling |
| 2 | http://arxiv.org/abs/2002.10616v1 | How many infections of COVID-19 there will be in the "Diamond Princess"-Predicted by a virus transmission model based on the simulation of crowd flow |
| 3 | http://arxiv.org/abs/2003.03149v1 | Space-time dependence of corona virus (COVID-19) outbreak |
| 4 | http://arxiv.org/abs/2003.06664v2 | Modelling and predicting the spatio-temporal spread of Coronavirus disease 2019 (COVID-19) in Italy |
| 5 | http://arxiv.org/abs/2003.06758v1 | Predicting COVID-19 distribution in Mexico through a discrete and time-dependent Markov chain and an SIR-like model |
| 6 | http://arxiv.org/abs/2003.07347v2 | Building a COVID-19 Vulnerability Index |
| 7 | http://arxiv.org/abs/2003.07778v1 | Day Level Forecasting for Coronavirus Disease (COVID-19) Spread: Analysis, Modeling and Recommendations |
| 8 | http://arxiv.org/abs/2003.09202v1 | New statistical model for misreported data with application to current public health challenges |
| 9 | http://arxiv.org/abs/2003.10891v2 | A simplified model for expected development of the SARS-CoV-2 (Corona) spread in Germany and US after social distancing |
| 10 | http://arxiv.org/abs/2003.11150v2 | Management strategies in a SEIR model of COVID 19 community spread |
| 11 | http://arxiv.org/abs/2003.13747v1 | The D model for deaths by COVID-19 |
| 12 | http://arxiv.org/abs/2003.13762v1 | Using VERA to explain the impact of social distancing on the spread of COVID-19 |
| 13 | 10.1101/2020.01.23.917351 | Pattern of early human-to-human transmission of Wuhan 2019-nCoV |
| 14 | 10.1101/2020.01.31.20019901 | Early dynamics of transmission and control of COVID-19: a mathematical modelling study |
| 15 | 10.1101/2020.02.09.20021444 | EPIDEMIC TRENDS ANALYSIS AND RISK ESTIMATION OF 2019-NCOV OUTBREAK |
| 16 | 10.1101/2020.02.14.20022913 | Estimating the Efficacy of Quarantine and Traffic Blockage for the Epidemic Caused by 2019-nCoV (COVID-19) |
| 17 | 10.1101/2020.02.14.20023127 | Substantial undocumented infection facilitates the rapid dissemination of novel coronavirus (COVID-19) |
| 18 | 10.1101/2020.02.16.20023614 | Utilize State Transition Matrix Model to Predict the Novel Corona Virus Infection Peak and Patient Distribution |
| 19 | 10.1101/2020.02.17.20023630 | Estimating the case fatality ratio of the COVID-19 epidemic in China |
| 20 | 10.1101/2020.02.17.20024018 | COVID-19 in a Designated Infectious Diseases Hospital Outside Hubei Province, China |
| 21 | 10.1101/2020.02.19.20025031 | A descriptive study of the impact of diseases control and prevention on the epidemics dynamics and clinical features of SARS-CoV-2 outbreak in Shanghai, lessons learned for metropolis epidemics prevention |
| 22 | 10.1101/2020.02.25.20027615 | Stochastic discrete epidemic modeling of COVID-19 transmission in the Province of Shaanxi incorporating public health intervention and case importation |
| 23 | 10.1101/2020.02.27.20028639 | Modeling the Epidemic Dynamics and Control of COVID-19 Outbreak in China |
| 24 | 10.1101/2020.03.03.20029843 | Effect of non-pharmaceutical interventions for containing the COVID-19 outbreak in China |
| 25 | 10.1101/2020.03.03.20030858 | Prediction of New Coronavirus Infection Based on a Modified SEIR Model |
| 26 | 10.1101/2020.03.12.20034595 | Rational evaluation of various epidemic models based on the COVID-19 data of China |
| 27 | 10.1101/2020.03.13.20035386 | Multi-city modeling of epidemics using spatial networks: Application to 2019-nCov (COVID-19) coronavirus in India |
| 28 | 10.1101/2020.03.15.20036707 | Temporal dynamics in viral shedding and transmissibility of COVID-19 |
| 29 | 10.1101/2020.03.17.20037770 | COVID-19 Progression Timeline and Effectiveness of Response-to-Spread Interventions across the United States |
| 30 | 10.1101/2020.03.18.20037473 | Now-casting the COVID-19 epidemic: The use case of Japan, March 2020 |
| 31 | 10.1101/2020.03.18.20037952 | Estimating Preventable COVID19 Infections Related to Elective Outpatient Surgery in Washington State: A Quantitative Model |
| 32 | 10.1101/2020.03.21.20040667 | COVID-19 in Canada: Predictions for the future and control lessons from Asia |
| 33 | 10.1101/2020.03.22.20041244 | Delaying the COVID-19 epidemic in Australia: Evaluating the effectiveness of international travel bans |
| 34 | 10.1101/2020.03.23.20041319 | Impact of changing case definitions for COVID-19 on the epidemic curve and transmission parameters in mainland China |
| 35 | 10.1101/2020.03.23.20042002 | Is a COVID19 Quarantine Justified in Chile or USA Right Now? |
| 36 | 10.1101/2020.03.24.20042374 | What is required to prevent a second major outbreak of the novel coronavirus SARS-CoV-2 upon lifting the metropolitan-wide quarantine of Wuhan city, China |
| 37 | 10.1101/2020.03.25.20043109 | Mitigation and herd immunity strategy for COVID-19 is likely to fail |
| 38 | 10.1101/2020.03.26.20044271 | Negligible Risk of the COVID-19 Resurgence Caused by Work Resuming in China (outside Hubei): a Statistical Probability Study |
| 39 | 10.1101/2020.03.29.20046565 | Eco-epidemiological assessment of the COVID-19 epidemic in China, January-February 2020 |
| 40 | 10.1101/2020.03.30.20040519 | Inferring Timing of Infection Using Within-host SARS-CoV-2 Infection Dynamics Model: Are "Imported Cases" Truly Imported? |
| 41 | 10.1101/2020.02.24.20026773 | Characterizing the transmission and identifying the control strategy for COVID-19 through epidemiological modeling |
| 42 | 10.21037/atm.2020.02.66 | Early estimation of the case fatality rate of COVID-19 in mainland China: a data-driven analysis. |
| 43 | 10.2807/1560-7917.ES.2020.25.10.2000180 | Estimating the asymptomatic proportion of coronavirus disease 2019 (COVID-19) cases on board the Diamond Princess cruise ship, Yokohama, Japan, 2020. |
| 44 | 10.2807/1560-7917.ES.2020.25.5.2000062 | Incubation period of 2019 novel coronavirus (2019-nCoV) infections among travelers from Wuhan, China, 20-28 January 2020. |
| 45 | 10.3201/eid2606.200357 | Serial Interval of COVID-19 among Publicly Reported Confirmed Cases. |
| 46 | 10.3390/ijerph17051679 | Prediction of Epidemic Spread of the 2019 Novel Coronavirus Driven by Spring Festival Transportation in China: A Population-Based Study. |
| 47 | 10.1186/s40249-020-00640-3 | A mathematical model for simulating the phase-based transmissibility of a novel coronavirus. |
| 48 | 10.1186/s41256-020-00137-4 | First two months of the 2019 Coronavirus Disease (COVID-19) epidemic in China: real-time surveillance and evaluation with a second derivative model. |

*Abbreviations:* DOI, digital object identifier
